# Hepatosplenic tuberculosis simulating secondary malignant lesions with cholangitis

**DOI:** 10.1186/s13104-016-2091-6

**Published:** 2016-06-20

**Authors:** Ibrahima Diallo, Ababacar Mbengue, Sara B. Gning, Mouhamed A. Amar, Bineta Ndiaye, Yankhoba Diop, Fatou Fall, Papa S. MBaye

**Affiliations:** Departement of Internal Medicine and Hepatogastroenterology, Hôpital Principal de Dakar, 1, Avenue Nelson Mandela, BP 3006 Dakar, Sénégal; Departement of Medical Imaging, Hôpital Principal de Dakar, Dakar, Sénégal; Anatomopathology Unit, Hôpital Principal de Dakar, Dakar, Sénégal

**Keywords:** Hepatic and splenic nodular lesions, Tuberculosis, Cholangitis, Acute hepatitis, Case report

## Abstract

**Background:**

Hepatic and/or splenic tuberculosis may simulate much pathology including malignancies, which can roam the diagnosis. Biopsy is necessary for diagnosis. The treatment allows healing and a cleaning of radiological lesions.

**Case presentation:**

We report a case of a 48-old-black Senegalese woman, immunocompetent, hospitalized for febrile jaundice and poor general condition. Imaging and hepatic biopsy showed hepatosplenic tuberculosis with cholangitis, simulating secondary malignancies lesions. The outcome was favorable under treatment.

**Conclusion:**

In front of hepatic nodular lesions simulating malignancies in a tuberculosis endemic areas, achieving a liver biopsy helps rectify the diagnosis.

## Background

Tuberculosis (TB) is endemic in our country. In the abdomen, the localization in the liver is rare, far behind the peritoneal and intestinal sites. Furthermore, tubercular cholangitis is rare. We report a case of hepatosplenic tuberculosis simulating secondary malignancies lesions associated with cholangitis in a multifocal tuberculosis context.

## Case report

A 48 years old-black Senegalese woman was hospitalized in June 2011 for cholestatic jaundice, right upper quadrant pain, fever and weak general condition with weight loss of 15 kg in 2 months. She had no medical or surgical history or pre-existing medical condition. On physical examination, she had jaundice and fever (38.5 °C). The abdomen was soft, painful to palpation of the right upper quadrant with hepatomegaly. She had no peripheral lymph nodes or splenomegaly. Laboratory investigations showed a discrete cytolysis with aspartate aminotransferase (AST) = 62 IU/l (2 N), alanine aminotransferase (ALT) = 49 IU/l (1,3 N), cholestasis with alkaline phosphatase (ALP) to 367 IU/l (2.7 N), GGT 291 IU/l (3.4 N), total bilirubin : 118.3 mg/l, with conjugated fraction to 77.3 mg/l, without liver deficiency [prothrombin time (PT) = 81 % albumin = 36 g/l]. There was an inflammation, with an erythrocyte sedimentation rate of 33 mm in the first hour, a serum fibrin to 4.1 g/l, and polyclonal hypergammaglobulinemia (31 g/l). The blood cell count and renal functions were normal. The alpha-fetoprotein levels were normal (3.7 ng/ml). Search of acid-fast bacilli by gastric aspirate was negative. The viral serology [hepatitis B virus (HBV), hepatitis C virus (HCV), human immunodeficiency virus (HIV), human T-lymphotropic virus 1 (HTLV1)] were negative. The urine cultures were sterile, without hematuria or pyuria.

**Fig. 1:**
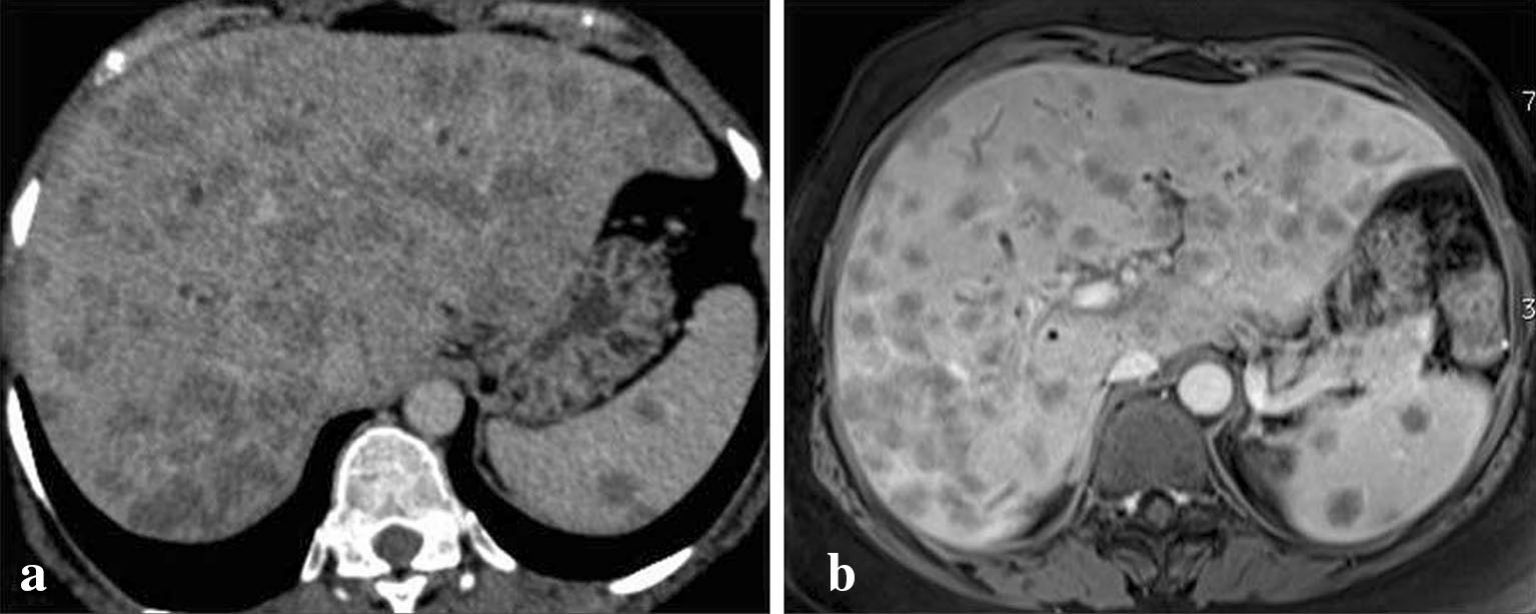
Multiple hepatic and splenic nodules hypodenses in the scanner (**a**) and hypointense on MRI with a delayed enhancement (**b**)

**Fig. 2:**
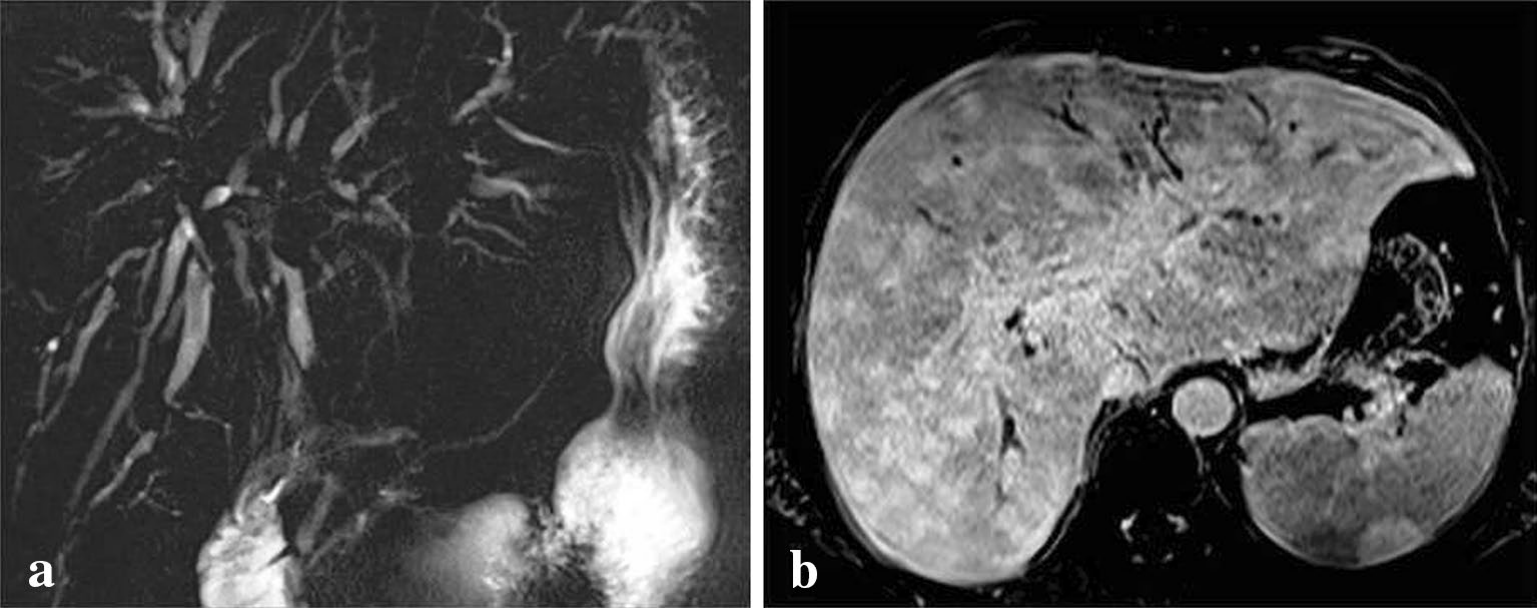
**a** Bile ducts amputation and **b** dilatation suggestive to cholangitis

**Fig. 3:**
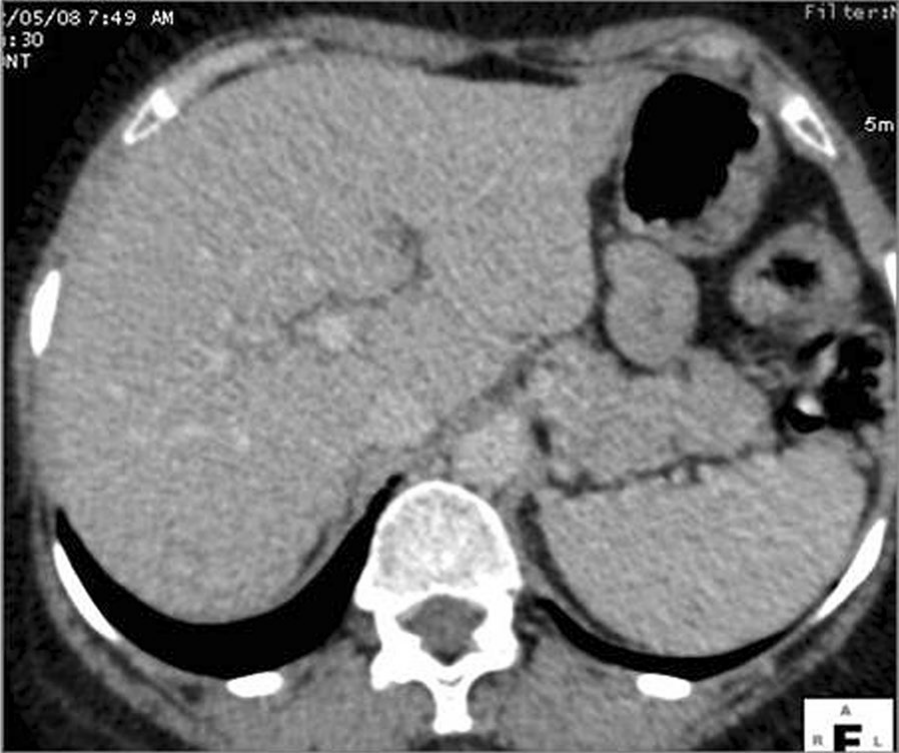
Control after 9 months of TB treatment

The chest x-ray was normal. Abdominal ultrasound showed a focal lesion of the left liver measuring 4 cm with metastatic appearance, several poorly defined hypoechoic areas, liver hilar lymphadenopathy and dilation of intrahepatic bile conduct. The thoraco-abdominal CT scan objectified multiple hypovascular nodules infiltrating the entire liver parenchyma (Fig. 1a, b), spleen nodules, pulmonary calcifications, mediastinal and abdominal lymph nodes. These nodules had a late enhancement of nonspecific appearance on Magnetic Resonance Imaging (MRI) with an amputation aspect of the biliary tree probably due to infiltration, realizing cholangitis (Fig. 2a, b). Liver biopsy showed hepatitis with reactional appearance with light activity and portal fibrosis without septa, cholestasis, epithelioid and giant cell granulomas without caseous necrosis. A multifocal tuberculosis (liver, bile ducts, spleen and lymph node) associated with cholangitis were diagnosed and treatment started with quadritherapy based on rifampicin (10 mg/kg/day), isoniazid (5 mg/kg/day), ethambutol (20 mg/kg/day) and pyrazinamide (30 mg/kg/day). A week later, the patient presented with severe acute drug hepatitis with ALT = 528 IU/L (13 N), AST = 973 IU/l (31 N) and PT 44 %, leading to treatment discontinuation. After normalization of these parameters after 10 days triple therapy combining rifampicin, ethambutol and levofloxacin was introduced with a good evolution: clinical improvement, normalization of liver function tests. After 4 months of triple therapy and 5 months of combination therapy (rifampicin and ethambutol), the outcome was favorable with disappearance of hepatic and splenic lesions on CT scan (Fig. 3).

## Discussion

Tuberculosis is a serious public health problem in our country. Abdominal localization ranks 4th behind lung, pleural and ganglion tuberculosis in our hospital [1]. Peritoneal TB is the most common abdominal form, followed by intestinal involvement. Isolated hepatobiliary TB is rare, due to the lack of oxygen in the liver, which is not favorable to the development of mycobacteria [2]. Liver infection can occur by hematogenous dissemination from the hepatic artery [3]. It can also go through the portal circulation, leading to macronodular, pseudotumoral or abscesses forms, often associated with intestinal localization [4]. It can be complicated with portal hypertension [5]. In our patient, the presence of hepatic and splenic macronodular lesions would be in favor of portal vein dissemination.

Clinical features usually associate hepatomegaly and/or splenomegaly with fever, dull pain in the right upper quadrant, jaundice and weak general condition, as in our patient. Exceptionally, ascites may be associated. However, these symptoms may be missing, especially in primitive forms. Hasan reported a case of isolated hepatosplenic TB without fever, shivering or jaundice, but only with abdominal pain and weight loss [6]. Disturbance of liver function tests resulting mostly to cholestasis and sometimes cytolysis. In our case, cytolysis was minimal and cholestasis marked. Liver tests can be normal despite the hepatic localization of TB [6].

In imaging, lesions are not specific [7]. In micronodular form, lesions are hypoechoic on ultrasound, punctate and hypodense on CT scan with less than 2 mm in size, scattered in the liver and the spleen. In macronodular forms, as described in our case, the ultrasound shows one or more masses with echogenicity and variable size, usually hypoechoic. In CT scan, the early lesions are isodense, become hypodense and eventually calcified. The contrast medium injection causes an annular enhancement [7]. On MRI, the most evocative aspect is a hyperintense lesion on the periphery, with lesser intensity at the center on T2-weighted sequences, and which enhances the periphery after contrast injection [7]. Spleen locations often associated, as in our case, in the form of hypoechoic/hypodense regular lesions, or irregular anechoic (abscess) or calcified nodule should be sought [8]. Furthermore imaging can show hepatic TB like a tumor mass as described by Kuçukmetin [9]. MRI also showed lesions suggestive of secondary sclerosing cholangitis in this context to tuberculosis. Tuberculous cholangitis is rare. This would be the cause of persistent jaundice in our patient. Imaging findings can vary from bile duct thickening, biliary dilatation and long and smooth strictures to frank obstructive biliopathy [7]. In the serie of Amarapurkar, 39.4 % of patients with hepatic TB, had biliary lesions, but only 5.2 % with biliary strictures, and the majority had biliary obstruction due to lymph node masses (31.5 %) [10]. In our patient, the bile ducts strictures were due to tuberculous infiltration.

The differential diagnosis will be made with secondary locations carcinoma, lymphoproliferative disorders and other granulomatosis. Diagnosis is based on histology through biopsy with a fine needle of the most accessible lesions. Epithelioid granuloma and giant cells are found in 80–100 % of cases, with caseous necrosis in 30–83 % of cases, or acid fast bacilli (AFB) in 59 % of cases [8]. PCR techniques detecting *Mycobacterium tuberculosis* may help the diagnosis [3]. The diagnosis of hepatic tuberculosis requires looking for immunosuppression. Our patient did not consume alcohol, was not diabetic, was not taking corticosteroids or immunosuppressive treatment and retroviral serology were negative to HIV and HTLV-1.

Treatment is based on TB medicines, according to different protocols. In Senegal, the quadruple therapy (isoniazid, rifampicin, éthambuthol pyrazinamide) for 2 months, followed by combination therapy (isoniazid, rifampicin) for 4 months, is the standard treatment. Some authors recommend prolonging treatment for 12 months [11]. In our case, the patient received conventional quadruple therapy which was stopped because of liver toxicity which can occur during treatment with a frequency ranging from 2 to 28 % [12]. Female gender, malnutrition, an existing liver disease, conditions met in our patient, are among others, factors favoring the occurrence of toxicity [12]. In our patient, it was decided to stop definitively isoniazid and pyrazinamide which are the most purveyors drugs hepatotoxicity, and introduce a second line anti-TB drugs (levofloxacin) associated with rifampicin and ethambutol, with good evolution. In hepatic or hepatosplenic tuberculosis, evolution is favorable in 67–100 % of cases with radiological healing and cleaning [10, 11] of lesions.

## Conclusions

Hepatic tuberculosis simulates many diseases, making diagnosis often difficult. Think about it in a suggestive clinical and epidemiological context. Imaging data is not specific and liver biopsy is always necessary. Evolution is often favorable if treatment is well conducted by monitoring the hepatotoxicity of antituberculosis drugs.

## Abbreviations

TB: tuberculosis
ALT: alanine aminotransferase
AST: aspartate aminotransferase
GGT: gamma glutamyl transferase
PT: prothrombin time
HBV: hepatitis B virus
HCV: hepatitis C virus
HIV: human immunodeficiency virus
HTLV1: human T-lymphotropic virus 1
CT scan: computed tomography scanning
MRI: magnetic resonance imaging
AFB: acid fast bacilli
PCR: polymerase chain reaction
